# Perceived quality of maternal care and its barriers based on women’s perspective in hospitals of Northwest Ethiopia: a qualitative study

**DOI:** 10.3389/fmed.2024.1387710

**Published:** 2024-10-23

**Authors:** Maru Mekie, Yismaw Yimam Belachew, Ermias Sisay Chanie, Wubet Alebachew Bayih, Setegn Muche Fenta, Gedefew Abeje Masresha, Wassie Yazie Ferede, Dagne Addisu

**Affiliations:** ^1^Department of Midwifery, College of Health Sciences, Debre Tabor University, Debre Tabor, Ethiopia; ^2^Department of Gynecology and Obstetrics, School of Medicine, College of Health Sciences, Debre Tabor University, Debre Tabor, Ethiopia; ^3^Department of Pediatrics and Child Health Nursing, College of Health Sciences, Debre Tabor University, Debre Tabor, Ethiopia; ^4^Department of Statistics, College of Natural and Computational Sciences, Debre Tabor University, Debre Tabor, Ethiopia; ^5^Department of Nursing and Midwifery, Debre Tabor Health Science College, Debre Tabor, Ethiopia

**Keywords:** quality, maternal care, perception, perceived barriers, Ethiopia

## Abstract

**Background:**

The Sustainable Development Goals (SDGs) in health focus on achieving equity to reduce maternal mortality disparities among subpopulations globally. The goal is to lower the maternal mortality ratio (MMR) to below 70 per 100,000 live births by 2030 for countries with an MMR below 420 in 2010. For those exceeding 420, the target is to keep the MMR below 140 by 2030. This target could not be achieved unless quality maternal health care service is implemented in all health care settings. This study aimed to assess the quality of maternal care and perceived barriers based on women’s perspectives among women who receive delivery services in hospitals of South Gondar Zone, Northwest, Ethiopia.

**Methods:**

A phenomenological method was implemented to explore women’s perspectives on quality maternal care and its perceived barriers. An in-depth interview (IDI) was used to collect data using a semi-structured in-depth interview guide. The study was conducted from January 1–30, 2021. All in-depth interview notes were translated into meaningful notes. Then notes were organized by coding texts into meaningful elements using open code version 4.03 software.

**Results:**

The majority (14 in 20) tried to interpret quality care in terms of respect during procedures, providing family support, and timely care. Some (6 in 20) tried to associate quality care with the competency of care providers, the cleanliness of the procedure, and privacy during care. With regards to perceived barriers to quality care, the majority (15 in 20) of the IDI participants agreed that a high caseload, limited drugs, and administrative problems were barriers to providing quality maternal care.

**Conclusion:**

Participants articulate a multifaceted understanding of quality maternal care that encompasses emotional support, accessibility, cleanliness, timely interventions, and respectful treatment. They also identify significant barriers that stem from facility limitations, provider attitudes and knowledge, and administrative inefficiencies. Addressing these barriers is essential for enhancing the quality of maternal health services.

## Introduction

According to the World Health Organization (WHO), quality care is defined as the extent to which health care services provided to individuals and patient populations improve desired health outcomes which are achieved through the provision of safe, effective, timely, efficient, equitable, and people-centered health care (1). Skilled professional care throughout pregnancy, childbirth, and the postpartum period is crucial for safeguarding the health of both mothers and newborns. Such interventions significantly lower the risks of complications, enhancing maternal well-being and ensuring safer deliveries, ultimately contributing to reduced rates of morbidity and mortality for both mothers and infants (2, 3).

The Sustainable development goals (SDGs) in health prioritize equity measures to eliminate disparities in maternal mortality between subpopulations within all countries. The SDG aims to decrease the maternal mortality ratio (MMR) to below 70 per 100,000 live births by 2030 in countries that had an MMR of less than 420 in 2010, representing a two-thirds reduction from the baseline. For countries with an MMR exceeding 420 in 2010, the target is to ensure that the MMR does not surpass 140 per 100,000 by 2030. Ethiopia falls into the second category, which cannot be achieved without the implementation of high-quality maternal health care services across all health care settings (4).

The WHO developed quality assessment guidelines to be implemented at a health facility level to improve pregnancy outcomes (5). Maternal morbidity and mortality can be prevented through nutritional intervention, access to contraceptive services, skilled delivery care, emergency as well as comprehensive obstetric care. Although the number of health facilities in Ethiopia has increased, they are still insufficient and unevenly distributed across various regions of the country (6–8).

There is low utilization of existing health care in Ethiopia and other developing countries due to poor health-seeking behavior which could be related to poor quality of care and low client satisfaction (7, 9, 10). Low-quality care at healthcare facilities can greatly impact client satisfaction, which in turn can discourage individuals from seeking necessary health services. Women who believe that the quality of care is inadequate are more likely to avoid accessing health services, even when life-saving interventions are available (6, 11–13). A study conducted in Zambia indicated that 54.2, 70.8, and 64.0% of women refuse to use antenatal care (ANC) due to experiences of poor service quality, unfriendly staff attitude, and unavailability of staffs, respectively (14). Like ways, an analysis of the Ethiopian Demographic and Health Survey (EDHS) revealed that 36, 43, and 21% of women received quality antenatal care (ANC), intrapartum care, and postnatal care (PNC) services, respectively. Similarly, a cross-sectional study carried out across various health facilities in Ethiopia found that the quality of the maternal and neonatal health output component was 48% (15, 16).

Studies indicated that poor quality of facility-based care for women and newborns is the major contributing factor to the increased rates of maternal and perinatal morbidity and mortality (6, 17). On the contrary, good service quality plays an important role in client satisfaction which ensures continued uptake of services (13). While assessing the quality of maternal care based on women’s perspective is crucial, there is a lack of information available in this area.

Effective communication and engagement among healthcare providers and other stakeholders are essential to ensure that health care services are responsive to women’s needs and preferences in all contexts and settings (10). Analysis of Nepal’s demographic and health survey indicated that only 38% of antenatal care clients were very satisfied with the services provided at health facilities (12). In the same manner, an analysis of national health system surveys in five African countries about the quality of basic maternity care found that primary health facilities had low-quality scores, particularly delivery services on the basic maternal care quality index (9). A study conducted in Northern Ethiopia indicated that only 29.2% of mothers received good quality care during intrapartum and immediate postpartum periods (18), though women’s perceptions of quality care have not been addressed in the study.

Assessing maternal health care quality from women’s perspectives is crucial for informing policymakers and stakeholders. This evaluation can guide necessary improvements, ultimately reducing maternal morbidity and mortality rates. This study aimed to assess the quality of maternal care and its perceived barriers based on women’s perspectives.

## Methods

### Study design, area, and period

A qualitative study was undertaken using a phenomenological technique to investigate the quality of maternal care based on the clients’ perspectives and perceived barriers to quality care. This study was conducted from January 1–30, 2021.

There are ten hospitals in the South Gondar Zone of the Amhara Region, including one Comprehensive Specialized Hospital and Nine Primary Hospitals. The study was conducted in three selected hospitals (Debre Tabor Comprehensive Specialized Hospital, Mekane Eyesus Primary Hospital, and Addis Zemen Primary Hospital). The three hospitals were selected for the study since they have high caseloads in maternal health services compared to other hospitals in the Zonal Administration. After selecting the hospitals, the researchers met in person with hospital officials to present a support letter, which would help establish contact with study participants.

### Study population and sampling technique

Women who received delivery services at the selected hospitals were included in the in-depth interview (IDI). For the sake of exploring the experience of care in terms of quality, those who delivered their last child at health facilities were purposefully selected for the study. This study included women who had normal spontaneous vaginal deliveries with normal birth outcomes. Twenty IDIs (5 in each of the two Primary Hospitals and 10 in the Referral Hospital) were carried out. The study utilized theoretical saturation to determine the appropriate sample size, ensuring that sufficient data were collected to effectively capture women’s perspectives on the quality of maternal health care.

### Data collection tool and data collection process

An interview guide prepared in the Amharic language was used for an IDI For data collection, three BSc midwives who had experience in qualitative data collection were recruited. A one-day training session was conducted for data collectors, focusing on essential skills for conducting interviews, taking field notes, and managing data for IDI to effectively gather and document information, ensuring a comprehensive understanding of the subject matter. The training was provided by the authors of the research project.

The face-to-face interview using a semi-structured, IDI guide was conducted in a separate and quiet room to maintain good focus. The interview was conducted within 6 h of delivery before discharge, and each IDI took 30–50 min. Aside from taking notes during the interview, there was a 10-min gap between each interview to have adequate time to take quotes in every interview. There was a daily meeting between the researchers and the data collector to discuss about the data collection process.

### Data quality assurance

Proper recording and abstraction of notes were carried out on filed work as well as transcription time by taking adequate time to maintain the quality of the data. For the sake of maintaining data quality, the data were triangulated by time, person, and place to maintain the credibility and dependability of the data.

Data triangulation was achieved by comparing the information collected at different times by the same individual, allowing for time-based triangulation throughout the study periods. Similarly, to triangulate data by person, daily debriefing sessions were held with both the data collectors and researchers. This process aimed to address errors and facilitate the sharing of experiences among data collectors during the data collection phase. Additionally, researchers provided in-person supervision throughout the data collection periods. Furthermore, triangulation of the collected data across three different hospitals was conducted daily during the study, serving as a method for triangulation by place.

### Data analysis

Qualitative content analysis was employed to analyze the qualitative data. The notes, originally collected in Amharic, were translated into English by two independent authors (the first and second authors). Following this, all in-depth interview notes were converted into meaningful notes. These translated notes were then reviewed multiple times by the remaining authors to ensure accuracy. After proofreading the translated notes, the data were organized by coding the texts into meaningful elements using Open Code Version 4.03 software, again by the two independent authors (the first and second authors). Difference in coding between the two authors were resolved by discussion through the involvement of other authors.

### Operational definition

Perceived quality: Perceived quality of maternal care as experienced by women reported using descriptive statistics.

Perceived barriers to quality care: Barriers to quality maternal care based on women’s view reported in narration.

### Ethics approval and consent to participate

The study received ethical approval from the research ethics committee of the College of Health Sciences at Debre Tabor University. Following this ethical approval, a support letter was obtained from the College. The research was conducted in accordance with the Helsinki Declaration after permission was sought from each hospital to carry out the study. The purpose of the interviews was explained to the individual IDI participants, and written informed consent was obtained from each participant before the interviews commenced.

## Results

This study aimed at assessing the quality of maternal care based on women’s perspectives among 20 purposefully selected participants in three selected hospitals in the South Gondar Zone.

### Characteristics of the IDI participants

Twenty women who visited the selected hospitals for delivery services participated in the IDI. Regarding the socio-demographic characteristics of the participants, 8 (40%), 5 (25%), and 4 (20%) were housewives, merchants, and government employees by occupation, respectively. The mean age of the IDI participants was 31.2 years, with an age range of 21–40 years (Table 1).

**Table 1:** Participants’ information and socio-demographic characteristics.

Code	Age	Occupation	Study Facility	Residence	Gravidity	Parity	Education Level
IDI01	28	Housewife	Facility1	Rural	III	II	No formal education
IDI02	30	Government employee	Facility1	Urban	II	I	College
IDI03	21	Housewife	Facility1	Rural	II	I	Primary
IDI04	25	Merchant	Facility1	Urban	II	I	Secondary
IDI05	33	Housewife	Facility1	Rural	III	II	No formal education
IDI06	36	Housewife	Facility1	Urban	III	II	College
IDI07	38	Government employee	Facility1	Urban	IV	II	College
IDI08	40	housewife	Facility1	Urban	V	IV	Primary
IDI09	22	Government employee	Facility1	Urban	II	I	College
IDI10	26	Housewife	Facility1	Rural	III	I	Primary
IDI11	24	Merchant	Facility2	Urban	II	I	Secondary
IDI12	29	Private employee	Facility2	Urban	II	I	College
IDI13	31	Housewife	Facility2	Rural	IV	III	No formal education
IDI14	37	Housewife	Facility2	Urban	III	II	Primary
IDI15	39	Merchant	Facility2	Urban	V	IV	College
IDI16	36	Merchant	Facility3	Urban	IV	III	Secondary
IDI17	27	Daily laborer	Facility3	Rural	II	I	Primary
IDI18	28	Merchant	Facility3	Urban	III	II	Secondary
IDI19	36	Government employee	Facility3	Urban	IV	III	College
IDI20	38	private employee	Facility3	Urban	V	IV	College

Facility1, Debre Tabor Comprehensive Specialized Hospital; Faclity2, Mekane Eyesus Primary Hospital; Facility3, Addis Zemen Primary Hospital.

### Clients’ perception of quality care

Quality maternal care was interpreted differently by the study participants. Most participants (14 out of 20) defined quality care in terms of respect during procedures, the provision of family support, and timely interventions. Some (6 out of 20) associated quality care with the competency of healthcare providers, the cleanliness of the environment, and the maintenance of privacy during care. Three main themes emerged from the IDIs: facility-related factors, provider-related factors, and the extent or process of care. Each main theme was further elaborated with two to six sub-themes.

### Facility-related quality of care

The study highlights critical facility-related themes impacting maternal care quality, focusing on equipment and structural issues. Most participants (13 out of 20) emphasized that quality care relies on the availability of essential drugs and medical equipment, noting significant shortages as a barrier. Concerns were raised about inadequate room ventilation and cleanliness, with some mothers forced to sleep on floors. Participants also pointed out the need for sufficient staff and privacy during examinations as indicators of quality care.

*“I do not exactly know what quality care is; I think it is about the availability of materials and drugs” (participant age 25 years old).*

*“When it comes to quality care in this hospital, I believe we are far from achieving it as it currently stands. How can we talk about quality care when there aren’t enough adequate rooms with clean and safe waiting beds? Mothers in labor and those who have just given birth often find themselves having to sleep on the floor” (participant, age 36).*

*“In my understanding, maternal care is considered high quality when there is an adequate number of staff, as well as access to ultrasound services, medications, and necessary drugs. Clients often find themselves having to pay for laboratory tests and medications outside the hospital” (participant, age 22).*

*“Maternal care is said to be quality when provided while maintaining the privacy of the patients. I have observed that the care providers exposed laboring mothers for examination without using covering materials, which might be discouraging in seeking care, especially for those who come from rural areas” (participant age 24 years old).*

### Professional-related quality of care

In the provider-related quality of maternal care, two subthemes have emerged. The themes were competency and the attitude of the professionals. Some (6 out of 20) of the IDI participants interpreted quality maternal care in terms of the competency and attitude of the providers. Most (13 out of 20) of the IDI participants believed that competency was not such a problem for professionals working in obstetrics and gynecology wards, but there was an attitude problem in some care providers that should be improved for improving clients’ satisfaction.

*“Maternal care is said to be quality if the service is provided by a component care provider. There should also be adequate equipment such as ultrasound and functioning laboratory settings since the availability of competent providers is not enough” (participant age 30).*

*“Quality care is care provided respectfully; most providers are not supportive during the labor process” (participant age 24).*

*“To me, maternal care is said to be quality if the care providers give information before and after doing a physical examination. a procedure. Most professionals do not give information about the progress of labor. Labor is stressful, and the woman as well as her family members may want information, but providers are usually not informative” (participant age 26 years old).*

### The extent of service based on clients’ perspective

Under the theme of service-related quality of care, six subthemes (aseptic technique, place of care, pain-free care, support during care, cost of care, and timely care) have emerged, which were agreed to be the main characteristics of quality care based on clients’ perspectives.

*“How do you say quality maternal care without having family members involved in the laboring process? Maternal care is said to be quality care when a family member is allowed to be present during the process of labor. Providers in this hospital do not allow our family member to be available in labor room” (participant age 38 years old).*

*“Maternal care is said to be quality care when the service is given free of charge. However, you are forced to spend money in one way or another when you visit hospitals during pregnancy” (participant age 39 years old).*

*“Quality care is a cost-free delivery service. It is clear that delivery service is a cost-free service; however, we are asked to buy drugs outside the hospital. I have asked to buy anti-D immunoglobulin outside the hospital” (participant age 29 years old).*

*“Quality care is all about providing care using very clean instruments and environments.” It may be either a high caseload or carelessness; the hospital rooms are not as clean as expected. As to my understanding, it is better to work on such issues” (participant age: 30 years old).*

*“I do not understand what quality care is. But for me, it may be about providing maternal health services in either one or two closely spaced areas. People who come for maternal health services from rural areas are usually moving here and there to search pharmacies and laboratories” (participant age 37 years old).*

*“Childbirth is a very painful event. Hence, providing anti-pain medication during labor is, in my view, evidence of quality care. It would be good if there were pain-free labor and delivery services” (participant age 36 years old).*

*“Quality care is all about providing timely care. There is a delay in providing care after reaching the health facility” (participant age 28).*

*“Quality care is shortening the duration of labor using medication. In my view, women should not stay longer in labor at the hospital level” (Participant age: 33 years old).*

### Perceived barriers to providing quality care

Concerning perceived barriers to providing quality maternal care, three themes; facility-related, provider-related, and administrative-related barriers have emerged. High caseloads, limited drugs, and administrative problems were stated as barriers to providing quality maternal care by the majority (15 in 20) of the participants.

### Facility related barriers

For facility-related themes, three sub-themes: - high caseload, equipment shortage, and limited numbers of staff have emerged. The majority (15 out of 20) of the IDI participants identified high caseloads, equipment shortages, and insufficient staff as key factors undermining quality maternal care, emphasizing the need for improvements in these areas.

*“As my understanding goes, a high caseload with limited beds might be the possible cause for gaps in providing quality care (participant age 36 years old).*

*“Drugs and medical equipment shortages may be the barriers to providing quality maternal care (participant age 22 years old).*

*“I have observed that there are no adequate numbers of staff at night, which results in a poor prognosis due to failure to maintain the timing of medication administration. Better to work on it” (participant age: 30 years old).*

### Provider related barriers

Attitude and knowledge gaps were subthemes that emerged from provider-related themes. Providers’ attitude and knowledge gaps hinder quality maternal care, with both provider and client knowledge deficiencies affecting respect and satisfaction in care provision.

*“Lack of respect may lead people to be unhappy with care provision, which affects the quality of the care” (participant age 24).*

*“Knowledge gaps on the provider side may affect the quality of care” (participant age 22 years old).*

*“Knowledge gaps on clients’ sides may affect the quality of care. Some women do not respect counseling from care providers (participant age 28 years old).*

### Administrative related barriers

In terms of administrative-related themes, two sub-themes (mistreatment and failure to purchase essential drugs) were discussed as possible factors that hinder quality care. Participants highlighted that administrative issues, such as delayed equipment purchases and salary payments, negatively impact care quality. Unhappy staff and lack of recognition for high performers further diminish performance and morale.

*“Administrative problems may affect the quality of care as they may not purchase equipment timely” (participant age 25 years old).*

*“If care providers are unhappy with the administrative body, the quality of work will be affected. So better to see such issues” (participant age 38 years old).*

*“Failure to pay salary and other related payments to the workers affects their performance and attitude, which directly affects the quality of care” (participant, age 36).*

*“Mistreatment or not recognizing high performers by the administrative body may affect the quality of care. Better to give recognition to improve the quality of care” (participant age 37 years old).*

## Discussion

In this study, we aimed to assess the quality of maternal care as well as perceived barriers to quality care based on clients’ perspectives. To deliver high-quality care, providers must recognize and respect clients’ needs, attitudes, and concerns. This understanding is essential for enhancing client satisfaction, ensuring ongoing service utilization, and achieving better health outcomes (19). Advocating for the highest attainable quality of care for every mother and newborn is imperative to achieving the SDGs since high maternal and neonatal mortalities are attributable to poor quality of care (20).

In this study, the majority (14 in 20) of the participants interpreted quality care in terms of respect during procedures, providing family support, adequate materials, and timely care. Some (6 in 20) tried to associate quality care with the competency of care providers, the cleanliness of the procedure, and privacy during care. Similar findings were reported in studies conducted in India and Malawi (11, 21). Similarly, a study conducted in Kenya demonstrated that facility and provider characteristics were important in shaping the quality of postpartum and intrapartum care (22).

With regards to facility-related quality of care, the availability of adequate drugs and medical equipment were stated as indicators of quality care in our study. Similar findings were reported in other studies (11, 21, 23). The finding of this study is also in agreement with WHO standards of quality care (5). Promote universal access to quality maternal and newborn care by ensuring the availability of high-impact interventions, essential medications, and necessary equipment. This approach aims to address equity gaps and enhance coverage for marginalized populations aside from improving the quality of maternal care (20). Hence, there is a need to avail adequate materials and drugs to provide quality maternal care which in turn improves maternal satisfaction and health-seeking behavior.

In this study, three themes—facility-related, provider-related, and administrative-related—were identified as barriers to providing quality maternal care from the women’s perspectives. Within the facility-related theme, three sub-themes emerged: high caseloads, equipment shortages, and insufficient staff. Our findings highlight an urgent need to enhance the availability of medical equipment, essential medications, and staffing levels to improve the quality of maternal care. Similar to our study, different barriers were found to affect the quality of maternal care in previous studies (18, 22, 24).

Similar to our study, inadequate medical equipment and essential medicines, a shortage of skilled staff, and limited capabilities to provide care were stated as barriers to providing quality maternal and child care in a qualitative study in low and middle-income settings (25). Similarly, a study conducted in India indicated that the availability of medicines, food, ambulance services, maintenance of cleanliness, privacy, positive client-provider interaction, and good and safe delivery with no complications were indicators of quality care based on the perspectives of women (11). Another study finding also reported shortages of resources as a barrier to accessing quality care in a global situational analysis through a meta-review (26).

In this study, the IDI participants stated knowledge and attitude-related gaps as barriers to providing quality maternal care. Similarly, providers’ attitudes and behaviors were reported to affect patients’ well-being, satisfaction, and case-seeking behavior in a systematic review and meta-analysis (27).

In terms of administrative-related barriers, two sub-themes (mistreatment and failure to purchase drugs) were discussed as possible factors that hinder the quality of maternal care. A similar finding was reported in a study conducted in Georgia, which reported disrespect and inequalities in accessing health care as barriers to receiving quality maternal care (28). We recommend that stakeholders, including the government, focus on enhancing respectful client interactions in maternity care and improving access to maternal health services at both primary and tertiary health facilities. Our study indicates a pressing need to improve the quality of maternal care to align with WHO standards, aiming to achieve the target of reducing maternal mortality to less than 70 per 100,000 live births by 2030. Additionally, it is essential that no country experiences a maternal mortality ratio exceeding 140 per 100,000 live births (4).

### Strengths and limitations of the study

The perceived quality of care as well as perceived barriers to quality maternal care were explored qualitatively in this study, which is the strength of the study. Understanding clients’ perspectives on quality care will be helpful to shape health programs in a user-friendly manner. Using In-depth interviews in a limited study setting might be the limitation of the study. We recommend future studies in this area using a large sample size and including as many health facilities as possible.

## Conclusion

In this study, we aimed to explore the quality of maternal care and its perceived barriers based on women’s perspectives. Participants articulate a multifaceted understanding of quality maternal care that encompasses emotional support, accessibility, cleanliness, timely interventions, and respectful treatment. They also identify significant barriers that stem from facility limitations, provider attitudes and knowledge, and administrative inefficiencies. Addressing these barriers is essential for enhancing the quality of maternal health services.

## Data Availability

The raw data supporting the conclusions of this article will be made available by the authors, without undue reservation.
